# Stephen Oroszlan and Retroviral Proteins

**DOI:** 10.3390/v14020290

**Published:** 2022-01-29

**Authors:** Alan Rein

**Affiliations:** HIV Dynamics and Replication Program, National Cancer Institute, Frederick, MD 21702, USA; reina@mail.nih.gov

Stephen Oroszlan received his early education in Hungary, graduating in 1950 from the Technical University in Budapest with a degree in chemical engineering. He worked for several years in Hungary before emigrating, via an arduous journey, to the United States in 1957. With assistance from the American Academy of Sciences, he obtained his Ph.D. in Pharmacology from Georgetown University School of Medicine. His first publications, beginning in 1964, dealt with the purification and properties of (as we now call them) retroviruses, and also other human viruses including adenoviruses and papillomaviruses, reflecting his support from the U.S. National Cancer Institute. After 1970, his research focused exclusively on retroviruses. The story of how he came to be associated with the Cancer Institute is told here [1] in a beautiful reminiscence by Ray Gilden, who played a major role in arranging this fortunate trajectory.

His research in this area was extraordinarily productive, resulting in over 200 publications between 1970 and 2006. It is characterized by a determination to describe the proteins in the viruses *specifically* and in *as much detail* as possible, and to see what can be learned from these descriptions. It is important to realize that sequencing nucleic acids was not routine then, as it is today. Moreover, a great deal of the information he obtained could not have been deduced from nucleic acid sequences. The present volume is a small collection of papers, either by his co-workers or by colleagues whose research was significantly influenced by his work. The breadth and diversity of these papers gives some indication of the remarkable ramifications of Steve’s work. 

Like many viruses, retroviruses synthesize many of their proteins in the form of polyprotein precursors, which are then cleaved to the final products by virus-coded proteases. One of the seminal contributions from Steve’s research is the isolation and characterization of viral proteases. This was, of course, crucial in the development of inhibitors of HIV-1 protease, which are among the principal weapons responsible for the success of highly active antiviral therapy in HIV-1-infected people. The present volume includes a review by Weber et al. [2] of research on HIV-1 protease. It also contains a research paper by Miczi et al. [3] describing a new assay for HIV-1 protease. Using biolayer interferometry, it generates quantitative data without the need for large quantities or purification of the enzyme. Sequences and three-dimensional structures of sites cleaved by HIV-1 protease during virus maturation are considered in fine detail in the review article by Swanstrom and Sundquist [4]. The issue also includes a paper by Mótyán et al. [5] on the ability of the protease to cleave sites within the NC protein. This work is in support of the proposal, still controversial, that the protease not only cleaves Gag in newly released virions, thus bringing about virus maturation, but also cleaves the mature NC protein in the course of infecting a new cell. We also present a detailed analysis by Alvarez et al. [6] on the mechanism of cleavage of HIV-1 RNA, an essential step in viral DNA synthesis, by the RNase H activity of reverse transcriptase.

Like those of many viruses, retroviral proteases are synthesized in ways which deviate from the canonical direct translation of mRNAs into the encoded proteins, and these unexpected mechanisms were obscure until the proteases were isolated and their amino-acid sequences determined. These unusual mechanisms have been collectively termed “recoding”, and the article here by Atkins et al. [7] is a comprehensive review of this topic, including a discussion of its implications and possible future applications. The contribution by Napthine et al. [8] is a characterization of a remarkable cellular protein, called “Shiftless”, that dramatically alters the efficiency of some recoding events. 

Much of the initial characterization of the proteins of HIV-1 and human T-cell leukemia virus, a retrovirus which induces leukemia, was performed by Steve’s lab in collaborations with that of Robert Gallo (in whose lab the virus was isolated) at the National Cancer Institute. The paper by Zella and Gallo [9] is a broad discussion of the mechanisms by which viruses and bacteria can induce cancer. 

My own research career was dramatically influenced by the discoveries in Steve’s lab, as I was repeatedly able to apply genetic and biological tools to further understand the functional significance of the phenomena his lab uncovered. My essay in the present volume tells four stories along these lines [10].

While HIV-1, the cause of the AIDS pandemic, was isolated in 1983 [11], the last two years have seen the appearance of a new virus causing a new pandemic: the coronavirus SARS-CoV-2, responsible for COVID-19. This virus, like retroviruses, undergoes proteolytic maturation, and Lockbaum et al. [12] report here the crystal structure of the main protease of SARS-CoV-2 in complex with a small-molecule inhibitor. One may hope that protease inhibitors will contribute to the defeat of COVID-19 in the not-too-far distant future.

One of Steve’s great pleasures in life was considering and discussing data and its implications. I have a vivid memory of him at his desk, poring over newly acquired amino-acid sequences; the sequence Pro-Pro-Pro-Tyr in murine leukemia virus p12 (later shown to be the Late domain of the virus [13]) elicited a loud exclamation of amazement, including a mild expletive. Figure 1 shows him at meetings, intensely engaged in discussion and enjoying the company of his colleagues. In my mind’s eye, I can see Steve beaming with pride at the many diverse outgrowths of his work described in this collection. It is a shame he did not live to see it.

## Figures and Tables

**Figure 1:**
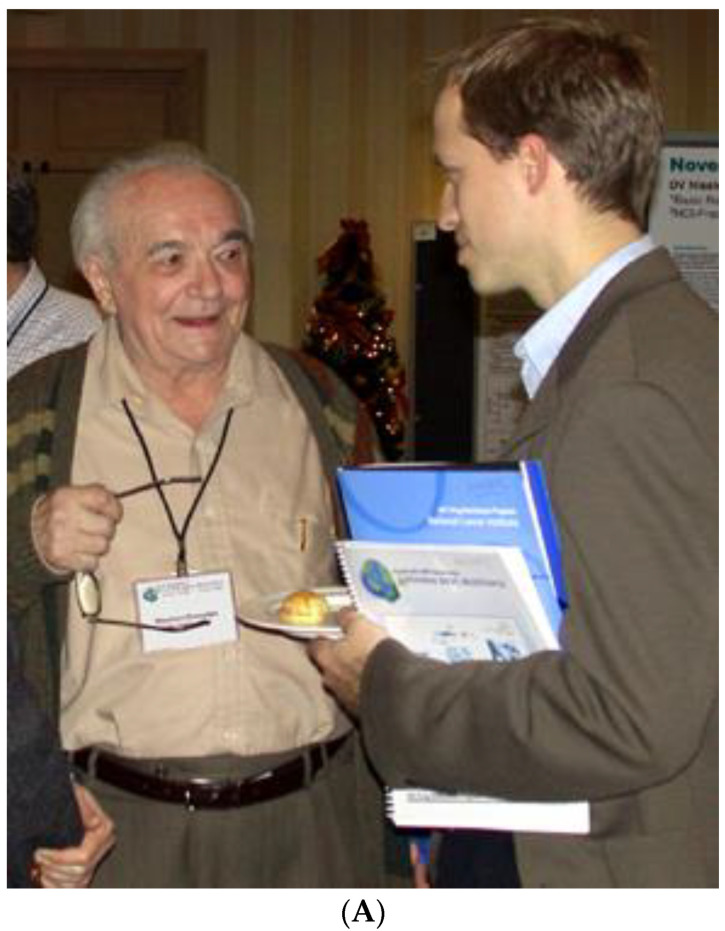

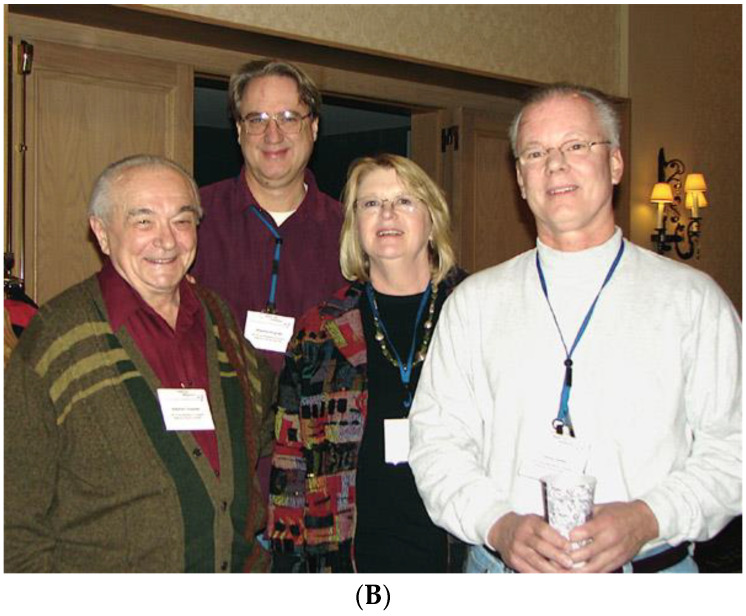
Steve Oroszlan in his element at meetings. (**A**) With Reuben Harris of the University of Minnesota; (**B**) with Steve Hughes, Gisela Heidecker, and Dave Derse, his colleagues at the NCI HIV Drug Resistance Program. Photos courtesy of Luis Menendez-Arias.
